# Effects of Lipid Emulsions in Parenteral Nutrition of Esophageal Cancer Surgical Patients Receiving Enteral Nutrition: A Comparative Analysis

**DOI:** 10.3390/nu6010111

**Published:** 2013-12-27

**Authors:** Wu-Ping Wang, Xiao-Long Yan, Yun-Feng Ni, Kang Guo, Chang-Kang Ke, Qing-Shu Cheng, Qiang Lu, Lan-Jun Zhang, Xiao-Fei Li

**Affiliations:** 1 Department of Thoracic Surgery, Tangdu Hospital, The Fourth Military Medical University, No.1, Xinsi road, Xi’an 710038, China; E-Mails: wwp._8@163.com (W.-P.W.); yanxiaolong@fmmu.edu.cn (X.-L.Y.); niyunfng@fmmu.edu.cn (Y.-F.N.); guokang91@126.com (K.G.); kechangkang@126.com (C.-K.K.); chechest@fmmu.edu.cn (Q.-S.C.); luqianglu@126.com (Q.L.); 2 Department of Thoracic Surgery, Cancer Center of Sun Yat-Sen University, State Key Laboratory in Southern China, No. 651, Dongfeng Donglu, Guangzhou 510060, China

**Keywords:** enteral nutrition, parenteral nutrition, olive oil, lipid emulsion, medium-chain triglyceride, long-chain triglyceride, immune function, esophageal cancer

## Abstract

***Background:*** Olive oil-based lipid emulsion (LE) and medium chain triglyceride/long chain triglyceride (MCT/LCT) emulsion are both LEs with low ω-6 polyunsaturated fat acids (PUFAs) content. However, which one of these LEs is associated with a lower infection risk in patients receiving parenteral nutrition (PN) remains unclear. The aim of the study was to compare the effects of the two LEs in PN in esophageal cancer patients undergoing surgery. ***Methods:*** Patients with resectable esophageal carcinoma were recruited and allocated randomly to two groups. The test group was given enteral nutrition (EN) with PN containing olive oil-based LE after tumor resection for ≥7 days, and the patients in the control group were supported by EN with MCT/LCT emulsion-based PN after surgery for the same time period. Immunological markers and inflammatory indicators were tested and perioperative clinical outcomes were determined. The trial was registered in the Chinese Clinical Trial Register, number ChiCTR-TRC-13003562. 94 Patients were recruited, and grouped (olive oil-based LE, *n =* 46 and MCT/LCT, *n =* 48), matched for sex, age, body mass index, histological type, TNM stage, and nutrition risk screening (NRS) 2002 score. ***Results:*** There were no differences in perioperative fever (>38 °C), infectious complications, length of hospital stay (>14 days), length of critical care stay (>2 days), time for oral food intake, and in-hospital mortality between the two groups. The test group showed a higher increase in IgG level compared with the MCT/LCT group (*p* = 0.028). There was no difference in other immunological markers and inflammatory indicators between the two groups. ***Conclusion:*** PN containing olive oil-based or MCT/LCT LEs had similar effects on perioperative outcome, cell-mediated immune function and inflammatory response in esophageal cancer patients who had undergone surgery and were receiving EN.

## 1. Introduction

Lipid emulsion (LE) is one of the most important components of parenteral nutrition (PN), and provides energy as well as essential fatty acids. LEs rectify the deficiency in old PN protocols that are characterized by hyperglycemia caused by a single energy source from glucose and an inadequacy of essential fatty acids [1,2]. However, LEs also have some variable biological effects due to different fatty acid composition or other components in different lipids [2,3,4,5,6,7]. Intralipid is the first long-chain LE based on soybean oil, and rich in ω-6 polyunsaturated fat acids (PUFAs) (e.g., linoleic acid). High ω-6 PUFA content produces too much pro-inflammatory eicosanoids, such as 2-series prostaglandins, 2-series thromboxanes and 4-series leukotrienes, which play important roles in the pro-inflammatory response and immunosuppression [7]. Many animal and clinical studies have shown that soybean oil-based LE induces oxidative stress, exaggerates the inflammatory response, hinders immune function, and increases the rate of infection [2,3,4,8,9,10,11].

To reduce the adverse effects of soybean oil-based LE, two strategies have been adopted. One is the advent of mixed LE with medium-chain triglyceride (MCT) and long-chain triglyceride (LCT), in which half of the LCT is replaced by MCT, which theoretically reduces the side effects arising from too much ω-6-PUFAs in LCT [4,12,13]. Another mixed LE is olive oil-based LE, which is composed of 80% olive oil and 20% soybean oil, in which the latter is sufficient to provide essential fat acids, but the ω-9 monounsaturated fatty acids in the olive oil exhibited no similar adverse effect as ω-6-PUFAs in soybean oil [2,4,5,12,14].

At present, both the olive oil-based LE and MCT/LCT LE are widely used for PN in China. However, few studies have focused on a comparison of the two LEs’ effects on inflammation and immunity. One *in vitro* study indicated that an olive oil-based LE was associated with bacterial recovery comparable to saline in the liver and lung rat model of systemic bacterial infection, while bacterial recovery rates from these organs were significantly higher for MCT/LCT and LCT [9]. In studies on neutrophil response [15,16,17,18], LEs inhibited calcium mobilization, a sign of cell activation, with emulsions including MCT having the greatest effect and olive oil–based LE the weakest effect [15,16,17]. Likewise LEs based on MCT/LCT or soybean oil influenced many other neutrophil responses, but olive oil-based LE was largely without effect [17,18]. In a study conducted in healthy volunteers, researchers found that MCT/LCT LE (500 mL given during 6 h) induced lymphocyte and neutrophil death [19]. In addition, a clinical study conducted in abdominal surgery patients showed that those patients who received olive oil-based LE had a lower level of pro-inflammatory cytokines, TNF-howed that than patients receiving MCT/LCT or soybean oil-based LE [20]. All these studies indicated that the olive oil-based LE might have less pro-inflammatory and immunosuppressive effects, and be associated with a lower infection risk in patients receiving PN than MCT/LCT.

Esophagectomy is a severely stressful operation characterized by cell-mediated immunosuppression preceded by a hyperinflammatory response, and with a high perioperative risk of infectious complications [11,21,22,23]. After esophagectomy, oral food intake is not allowed immediately, and EN combined with PN plays a key role in promoting patient recovery [24,25].

To our knowledge, there is no study comparing the use of olive oil-based LE with MCT/LCT LE in esophageal cancer patients. We hypothesized that olive oil-based LE might be a better alternative with a lower perioperative infection risk than MCT/LCT, and designed the present study to investigate the differences in the two LEs with regard to their effects on clinical outcome, immune function and inflammatory response in esophageal cancer patients who had undergone surgery and were receiving EN.

## 2. Material and Methods

### 2.1. Study Design and Patients

This prospective, double-blind controlled clinical trial randomized 94 patients (aged 35–70 years) with resectable esophageal cancer, to receive EN combined with PN containing olive oil-based LE or MCT/LCT LE after surgery for >7 days. All patients required radical esophagectomy with three-field lymph node dissection for esophageal carcinoma.

### 2.2. Exclusion Criteria

Exclusion criteria were as follows: (1) patients had participated in drug trial within 4 weeks of the present study; (2) LE was infused before surgery; (3) life expectancy < 7 days; (4) contraindications for PN (e.g., disturbance of blood coagulation, severe metabolic disease); (5) pregnancy or breastfeeding; (6) patients were potentially uncooperative or did not comply with the protocol; (7) severe cardiopulmonary insufficiency; (8) severe dyslipidemia [triglyceride or cholesterol levels > 2 times the upper limit of normal (ULN)]; (9) patients diagnosed with diabetes before surgery; (10) liver dysfunction (alanine/aspartate transaminase level > 3 times ULN, severe cholestasis, or conjugated bilirubin level > 2 times ULN); (11) chronic renal failure (blood urea nitrogen and creatinine > 2 times ULN); (12) allergic to any ingredients or accessories of LE (e.g., egg or soybean protein); (13) history of corticosteroids and immunosuppressive agents; (14) history of preoperative radiotherapy or chemotherapy; (15) history of autoimmune disease (e.g., rheumatoid arthritis); or (16) chronic diseases within 3 mo of the present study, which had a detrimental effect on the immune system, such as chronic infection. In addition, exit criteria were: (1) severe bleeding, pancreatic injury, cardiac arrest during operation; (2) active bleeding, pulmonary infarction, cerebrovascular accident, myocardial infarction, acute heart failure in perioperative period; or (3) the patient asked to leave the study. When the patients exited from the study, researchers had to make the final assessment, and document the early termination in the case report form. Strict follow-up had to be performed for patients who quit the study.

The study was conducted at Tangdu Hospital, a major teaching hospital affiliated with the Fourth Military Medical University and at Sun Yat-Sen University Cancer center in Southern China. Informed consent was obtained prior to randomization from study participants and the study was approved by Tangdu Hospital Ethics Committee at the Fourth Military Medical University. A research pharmacist at each institution coordinated treatment assignment following a computer-generated randomization table.

### 2.3. Surgical Approach and Process

Postoperative nutrition support for esophageal cancer patients was achieved through early EN combined with PN. The nutritional goal was 25~30 kcal/kg in both groups. A PN channel through the internal jugular vein or subclavian vein was established during anesthesia, and a nasoduodenal feeding tube was placed at operation when finishing anastomosis. EN was started with an initial dose of 200–250 mL of oligomeric or polymeric formula under 20 mL/h from nasoduodenal tube within 24 h after esophagectomy. EN dose was gradually increased every 12–24 h if there were no problems related to EN, and reached a maximum dose 6–7 days after starting EN in both groups. PN was started on the next day after esophagectomy and lasted for >7 days. Apart from the different LEs (20% olive oil-based LE, 250 mL *vs.* 20% MCT/LCT LE, 250 mL) (Table 1), PN in the two groups had the same ingredients: glucose, amino acids, glutamine, electrolytes, multivitamins and trace elements. We dispensed the PN using a double chamber bag (Baxter Healthcare) with a total volume of 3 L. We infused the PN solution by an all-in-one method through the internal jugular or subclavian vein once daily, with total energy provided by PN of 17.5 kcal/kg/day (provided by glucose and lipid). For an average weight of 60 kg, the patients received 1050 kcal total energy, 150 g glucose, 50 g lipid from PN, and the glucose energy *vs.* lipid energy rate was 16:9. The glucose *vs.* insulin rate was 4:1. No other immunonutrition was added to PN. This nutritional prescription was designed according to ESPEN Guidelines on PN for surgery [26] or intensive care [27] and met the basic needs for energy and liquid. We also included the same preventive antibiotics in the two groups. After 7 days PN, oral food intake was allowed on day 8 for patients with no anastomosis leakage or other complications affecting oral feeding. Otherwise, oral feeding was delayed according to the individual situation. Liver and renal function was measured at regular intervals.

The primary endpoint of the study was the number of infectious complications (pneumonia, bacteremia, wound infection, central venous catheter-related (CVCR) infection, urinary tract infection and anastomosis leakage), as described previously [28]. Secondary endpoints were other clinical outcomes, including perioperative fever (>38 °C), length of hospital stay > 14 days, critical care stay > 2 days, in-hospital mortality, oral food intake at day 8, immunological markers, and inflammatory indicators.

**Table 1:** The compositions of the two lipid emulsions.

Compositions	Olive oil based LE	MCT/LCT
Soybean oil (g/L)	40	100
Olive oil (g/L)	160	0
MCT (g/L)	0	100
Phospholipid (g/L)	12	12
Glycerol (g/L)	22.5	25
Energy (kcal/L)	2000	1908
Osmolarity (mosm/L)	270	380
PH	7–8	6.5–8.5
Saturated fatty acids (SFA) (%)	15	60
Mon-unsaturated fatty acids (MUFA) (%)	65	10
Essential poly-unsaturated fatty acids (EPUFA) (%)	20	30
ω-6 PUFAs:ω-3 PUFAs	9:1	8:1

### 2.4. Blood Sampling and Laboratory Methods

Blood samples were taken in the morning for every candidate at day −1 (the day before the operation), day 1 (the first day after the operation) and day 8 (the eighth day after the operation). Samples were collected in a preservation solution and stored at −80 °C. Routine blood tests, including lymphocyte count, serum IgA, IgG, and IgM, and complement components C3 and C4, and C reactive protein (CRP) were conducted in the clinical laboratory of Tangdu Hospital or Sun Yat-Sen University Cancer Center. Lymphocyte subpopulation percentage analysis (CD_3_^+^, CD_4_^+^, CD_8_^+^, and CD_4_^+^/CD_8_^+^) was performed through flow cytometry. The fluorescein-labeled antibodies were purchased from BD (Franklin Lakes, NJ, USA), the flow cytometer was from Beckman-Coulter (Fullerton, CA, USA). Interleukin (IL)-6 and tumor necrosis factor (TNF)-α were detected using a human IL-6 ELISA Kit (R & D Systems, Indianapolis, IN, USA) and human TNF-α ELISA Kit (BioSource, Nivelles, Belgium).

### 2.5. Statistical Analysis

A power analysis was conducted prior to the study based on preliminary data from Tangdu Hospital, which indicated an overall infection rate of 40% during EN combined with PN in patients with esophageal cancer undergoing esophagectomy with three-field lymph node dissection. Using an approximate two-sample proportion test, two-sided and α = 0.05, we estimated >80% power with 47 patients per group in order to detect a difference of >0.25 (the effect size of difference in outcome was estimated according to the previous literatures [29,30] and our clinical experience, and was considered clinically significant) in the occurrence rates for total infection between the two LEs study groups. Results are presented as mean ± SD. All data were analyzed using SPSS version 16.0 software (SPSS Inc., Chicago, IL, USA). The χ*^2^* test (or Fisher’s exact test) was used to compare clinical characteristics and clinical outcomes in the two groups, while the *t* test or Wilcoxon test and Mann-Whitney U test were used to compare the other tested parameters. *p* < 0.05 was considered statistically significant.

## 3. Results

### 3.1. Patients

From August 2012 to August 2013, 94 patients with resectable esophageal cancer were enrolled in our study: 46 in the olive oil-based LE group with an average age of 57.75 ± 7.77 years, and 48 in the MCT/LCT group with an average age of 57.80 ± 8.32 years. The two groups were matched well for baseline characteristics including sex, age body mass index (BMI), histological type, TNM stage, and nutrition risk screening (NRS) 2002 score (Table 2). Both emulsions were well tolerated by all patients, and no one left the study due to adverse reactions.

**Table 2:** Basic clinical characteristics in the two groups.

Characteristics	Olive oil-based LE(*n* = 46)	MCT/LCT(*n* = 48)	*p*
**Sex**			0.721 *
Male	34	37	
Female	12	11	
**Age (y)**	57.75 ± 7.77	57.80 ± 8.32	0.980 ^†^
**BMI** (kg/m^2^)	22.12 ± 2.98	21.45 ± 2.97	0.362 ^†^
**Histological type**			0.532 *
Squamous cell	36	40	
Adenocarcinoma	10	8	
**TNM stage**			0.717 ^§^
I	1	3	
II	15	17	
III	30	28	
IV	0	0	
**NRS 2002 score**			0.840 *
1	7	8	
2	20	18	
≥3	19	22	

Note: * Pearson χ^2^ test, ^†^ *t* test, ^§^ Fisher’s exact test.

### 3.2. Clinical Outcomes

With regard to the clinical outcomes, no differences were found in perioperative fever (>38 °C), total infection, pneumonia, bacteremia, wound infection, CVCR infection, urinary tract infection, anastomosis leakage, length of critical care stay (>2 days), length of hospital stay (>14 days), time of oral food intake, and in-hospital mortality between the two groups (all *p* > 0.05, Table 3).

### 3.3. Analysis of Immune Function

Comparison of blood immunoglobulins and complement showed that IgA, IgG and IgM decreased significantly from day −1 to day 1, while C3 and C4 remained stable from day −1 to day 1. From day 1 to day 8, the IgA, IgG, IgM and C3 level increased in both groups. C4 level on day 8 remained stable in the MCT/LCT group but increased in the olive oil-based LE group. Although there was no significant difference in the changes in IgA, IgM, C3 and C4 between the two groups after 7 days PN, there was a more significant increase in IgG level from day 1 to day 8 in the olive oil-based LE group compared with the MCT/LCT group (Table 4).

**Table 3:** Clinical outcomes in patients treated with PN containing olive oil-based LE or MCT/LCT, *n* (%).

Clinical outcomes	Olive oil-based LE (*n* = 46)	MCT/LCT (*n* = 48)	*p*
Perioperative fever (>38 °C)	29(63)	34(71)	0.422 *
Total infection patients	21(46)	25(52)	0.533 *
Pneumonia	5(11)	7(15)	0.590 *
Bacteremia	3(7)	3(6)	>0.99 ^†^
Wound infection	8(17)	9(19)	0.864 *
CVCR infection	3(7)	2(4)	0.674 ^†^
Urinary tract infection	2(4)	3(6)	>0.99 ^†^
Anastomosis leakage	6(13)	4(8)	0.519 ^†^
Critical care stay (>2 days)	15(32)	18(38)	0.619 *
Oral food intake at day 8	27(59)	31(65)	0.557 *
Length of hospital stay (>14 days)	23(50)	27(56)	0.544 *
In-hospital mortality	2(4)	1(2)	0.613 ^†^

Note: * Pearson χ^2^ test, ^†^ Fisher’s exact test.

**Table 4:** Comparison of immunoglobulins and complement between the two groups.

Immunoglobulins and complement	Day −1	Day 1	Day 8	*p*
**IgA** (g/L)				0.867 ^§^
Olive oil-based LE	2.14 ± 0.96	1.90 ± 0.95 *^,†^	2.57 ± 1.11	
MCT/LCT	2.29 ± 1.01	1.84 ± 0.88 *^,†^	2.69 ± 1.72	
**IgG** (g/L)				0.028 ^§^
Olive oil-based LE	11.1 ± 2.76	9.18 ± 2.93 *^,†^	12.2 ± 3.79	
MCT/LCT	12.39 ± 3.57	9.10 ± 2.30 *^,†^	11.02 ± 2.82	
**IgM** (g/L)				0.767 ^§^
Olive oil-based LE	0.97 ± 0.45	0.92 ± 0.65 *^,†^	1.87 ± 1.83	
MCT/LCT	1.78 ± 2.25	1.06 ± 0.68 *^,†^	1.60 ± 0.81	
**C3** (g/L)				0.547 ^§^
Olive oil-based LE	1.25 ± 0.20	1.33 ± 0.25 ^†^	1.50 ± 0.27	
MCT/LCT	1.24 ± 0.21	1.26 ± 0.23 ^†^	1.40 ± 0.22	
**C4** (g/L)				0.198 ^§^
Olive oil-based LE	0.31 ± 0.09	0.36 ± 0.28 ^†^	0.39 ± 0.18	
MCT/LCT	0.40 ± 0.38	0.33 ± 0.15	1.34 ± 0.16	

Note: * day −1 *vs.* day 1, *p* < 0.05. ^†^ day 1 *vs.* day 8, *p* < 0.05; ^§^ *p* value of olive oil-based LE *vs.* MCT/LCT change from day 1 to day 8.

Lymphocyte count and CD_8_^+^ subpopulation decreased significantly on postoperative day 1 in both groups, while CD_3_^+^, CD_4_^+^, and CD_4_^+^/CD_8_^+^ ratio remained stable at postoperative day 1 in both groups. When compared with day 1, all lymphocyte subpopulations remained stable at day 8 in both groups. The change in lymphocyte subpopulations did not differ significantly between the two groups (all *p* > 0.05, Table 5).

**Table 5:** Comparison of lymphocyte count and subpopulations between the two groups.

Lymphocyte	Day −1	Day 1	Day 8	*p*
**Lymphocyte count (×10^9^)**				0.977 ^§^
Olive oil-based LE	1.85 ± 0.58	1.45 ± 1.89 *	1.73 ± 1.54	
MCT/LCT	1.86 ± 0.56	0.98 ± 0.52 *^,†^	1.33 ± 0.54	
**CD_3_^+^** (%)				0.576 ^§^
Olive oil-based LE	59.38 ± 14.58	51.65 ± 16.63	51.06 ± 21.10	
MCT/LCT	51.54 ± 27.94	41.64 ± 23.07	41.33 ± 26.46	
**CD_4_^+^** (%)				0.278 ^§^
Olive oil-based LE	34.65 ± 10.39	30.27 ± 15.38	31.39 ± 14.25	
MCT/LCT	33.21 ± 18.52	30.69 ± 17.14	27.81 ± 18.94	
**CD_8_^+^** (%)				0.642 ^§^
Olive oil-based LE	35.31 ± 12.36	26.83 ± 12.07 *	25.18 ± 12.91	
MCT/LCT	23.95 ± 12.22	18.42 ± 9.36 *	17.39 ± 11.18	
**CD_4_^+^/CD_8_^+^** (%)				0.312 ^§^
Olive oil-based LE	0.92 ± 0.97	0.91 ± 1.43	0.58 ± 0.24	
MCT/LCT	2.26 ± 4.92	2.01 ± 3.78	2.21 ± 4.63	

**N**ote: * day −1 *vs.* day 1, *p* < 0.05. ^†^ day 1 *vs.* day 8, *p* < 0.05; ^§^ *p* value of olive oil-based LE *vs.* MCT/LCT change from day 1 to day 8.

### 3.4. Analysis of Inflammatory Response

IL-6 level in the olive oil-based LE group and TNF-α in the MCT/LCT group both increased on postoperative day 1. Both IL-6 and TNF-α remained stable at day 8 when compared with day 1. There was also no difference in the changes in cytokines from day 1 to day 8 between the two groups. Comparison of C-reactive protein (CRP) revealed a rapid increase at day 1 after esophagectomy (*p* < 0.001), and there was a significant decrease at day 8 compared with day 1 after 7 days PN in both groups (*p* < 0.001). There was no significant difference in the change in CRP between the two groups (Table 6).

**Table 6:** Comparison of proinflammatory indicators between the two groups.

Cytokines	Day 1	Day 1	Day 8	*p*
**IL-6** (pg/mL)				0.923 ^§^
Olive oil-based LE	47.08 ± 26.60	61.78 ± 29.13 *	61.56 ± 34.78	
MCT/LCT	59.53 ± 57.58	63.87 ± 30.02	68.94 ± 53.04	
**TNF-α** (pg/mL)				0.618 ^§^
Olive oil-based LE	54.62 ± 43.50	60.53 ± 33.14	67.05 ± 40.61	
**TNF-α** (pg/mL)				0.618 ^§^
MCT/LCT	57.16 ± 41.91	71.97 ± 32.58 *	75.25 ± 62.45	
**CRP** (mg/L)				0.704 ^§^
Olive oil-based LE	8.55 ± 17.04	160.56 ± 105.86 *^,†^	50.68 ± 59.60	
MCT/LCT	4.20 ± 5.81	152.82 ± 88.09 *^,†^	53.53 ± 73.96	

Note: * day −1 *vs.* day 1, *p* < 0.05. ^†^ day 1 *vs*. day 8, *p* < 0.05; ^§^ *p* value of olive oil-based LE *vs.* MCT/LCT change from day 1 to day 8.

## 4. Discussion

This prospective, double-blind randomized trial aimed compared differences in clinical outcomes in surgical esophageal cancer patients receiving EN combined with PN containing olive-oil-based or MCT/LCT LEs. We observed similar rates of infectious complications, and no significant differences in peri-operative fever (>38 °C), length of hospital stay, length of critical care stay (>2 days), in-hospital mortality, and time for oral food intake in esophageal cancer patients who had undergone surgery and were receiving EN. In addition, apart from the test group showing a greater increase in IgG level than the MCT/LCT group did, we detected little difference in immunological markers and inflammatory indicators between the two groups with PN containing different LEs.

Nutritional support for esophageal cancer patients undergoing surgery is one of the main indications for PN [24,25]. However, recent randomized trials and meta-analyses have suggested that treatment with PN may increase the risk for infectious complications and hospital mortality [31,32,33]. The increased rate of PN-associated complications is likely multifactorial, but may be related to immunological suppression or proinflammatory response of specific PN components, including administration of conventional soybean oil-based LE, which contains a high content of linoleic acid and ω-6 PUFAs [2,4,5,6,7,8,9,10]. Some studies have attributed their effect partially to the different fatty acid composition of the LEs [34], and that fatty acid constituents of parenteral LEs modify the immune response via alterations in membrane structure and fluidity, eicosanoid production, cell signaling, and gene expression [7]. Olive oil-based LE and MCT/LCT are well-known LEs designed to decrease the adverse effects of soybean oil-based LE [4,12]. Intravenous infusion of these two lipids may lead to a milder effect on the immune system.

However, the evidence is insufficient to define clearly the role of MCT/LCT with regard to its immunological effects. A meta-analysis summarized the published data before 2006, and found that none of the lipid regimens, including MCT/LCT, showed a clear effect on the evolution of immunological status in humans [12]. In our research, after the administration of MCT/LCT LE in PN for 7 days, lymphocyte count increased significantly. This is in line with the previous two studies suggesting that MCT/LCT emulsions are superior to 100% LCT emulsions, by having an immuno-restorative effect with regard to lymphocyte function [35,36]. However, in contrast, in a clinical study in healthy humans, intravenous infusion of an MCT/LCT LE was associated with decreased lymphocyte count [37]. Unlike MCT/LCT, the immunological neutrality of olive-oil-based LE has been confirmed by preclinical and clinical studies, with little effect on immune cell function [3,38,39]. One *in vitro* study showed that the oleic acid-rich olive oil-based LE was associated with less lymphocyte apoptosis, necrosis and DNA fragmentation compared with linoleic acid rich in LCT [38]. Another in vitro study showed that upregulation of activated antigens involved in T cell proliferation was enacted by LCT, but unaffected by olive oil-based LE [3]. Buenestado *et al.*, [18] showed that olive oil-based LE had no effect on lymphocytes and little effect on neutrophil function and leukocyte endothelial cell interaction. In our study, there was no change in lymphocyte count and subpopulation percentage in patients given PN containing olive oil-based LE for 7 days, which supports the minor effect of olive oil-based LE on cell-mediated immunity. However, we also measured immunoglobulins and complement, which reflect the humoral immune response [39]. After 7 days PN, IgA, IgG, IgM and C3 levels increased significantly in both groups, suggesting that both LEs promote the recovery of humoral immunity in patients undergoing esophagectomy. In addition, it was particularly interesting that the IgG level increased more significantly in patients receiving olive oil-based LE compared with MCT/LCT. This indicates that olive oil-based LE has greater activity in promoting recovery of humoral immunity. However, our results are insufficient to support the view that olive oil-based LE induces less cell-mediated immunosuppression than MCT/LCT emulsion.

With regard to the proinflammatory response, both LEs had a neutral effect on TNF-α, IL-6 and CRP in our study. In our opinion, this can be attributed to a decreased ω-6 PUFA content in olive oil-based LE and MCT/LCT LE. The ω-6 PUFA in soybean oil was associated with production of proinflammatory eicosanoids, which tend to regulate additional inflammatory mediators [40]. Two studies support this view [20,41]. One study demonstrated that total production of TNF-α increased significantly after receiving soybean oil-based LE, although there was no change in the MCT/LCT group [41]. The other study in abdominal surgery patients found that those who received olive oil-based LE had a low level of proinflammatory cytokines, TNF-α and IL-6 when compared with patients receiving MCT/LCT or soybean oil-based LE [20]. However, unlike the study by Demirer *et al.*, [20], there was no difference in the effects of olive oil-based LE and MCT/LCT LE on proinflammatory cytokines in our study.

The primary endpoint of our study was overall infection rate and the data showed no difference between the two LEs for infection rate. This was mainly due to similar effects on the immune system and proinflammatory response for the two LEs. The main reason may have been the relatively small number of patients enrolled in our study. Nonetheless, our study was unique in that it was, to the best of our knowledge, the first rigorous, double-blind trial to compare infection rates and other clinical outcomes and inflammatory and immune parameters after PN containing olive oil-based and MCT/LCT-based LE in esophageal cancer patients after surgery.

## 5. Conclusions

In conclusion, our study indicated that administration of EN combined with PN containing olive oil-based and MCT/LCT-based LE had similar effects on perioperative clinical outcomes, cell-mediated immunity, and proinflammatory response in esophageal cancer patients who had undergone surgery and were receiving EN. Further clinical trials with large numbers of patients are needed to clarify the potential advantage of olive oil-based LE on the humoral immune system.
